# Lactobacilli Reduce Chemokine IL-8 Production in Response to TNF-****α**** and *Salmonella* Challenge of Caco-2 Cells

**DOI:** 10.1155/2013/925219

**Published:** 2013-12-28

**Authors:** Da-Yong Ren, Chang Li, Yan-Qing Qin, Rong-Lan Yin, Shou-Wen Du, Fei Ye, Hong-Feng Liu, Mao-Peng Wang, Yang Sun, Xiao Li, Ming-Yao Tian, Ning-Yi Jin

**Affiliations:** ^1^Key Laboratory of Jilin Province for Zoonosis Prevention and Control, Institute of Military Veterinary, Academy of Military Medical Sciences, Changchun 130122, China; ^2^College of Food Science and Engineering, Jilin Agricultural University, Changchun 130118, China; ^3^Academy of Animal Science and Veterinary Medicine of Jilin Province, Changchun 130062, China

## Abstract

The probiotic properties of two selected lactobacilli strains were assessed. *L. salivarius* and *L. plantarum* displayed higher hydrophobicity (48% and 54%, resp.) and coaggregation ability with four pathogens (from 7.9% to 57.5%). *L. salivarius* and *L. plantarum* had good inhibitory effects on *S. aureus* (38.2% and 49.5%, resp.) attachment to Caco-2 cells. Live lactobacilli strains and their conditioned media effectively inhibited IL-8 production (<14.6 pg/mL) in TNF-**α**-induced Caco-2 cells. Antibiotic-treated and the sonicated lactobacilli also maintained inhibitory effects (IL-8 production from 5.0 to 36.3 pg/mL); however, the heat-treated lactobacilli lost their inhibitory effects (IL-8 production from 130.2 to 161.0 pg/mL). These results suggest that both the structural components and the soluble cellular content of lactobacilli have anti-inflammatory effects. We also found that pretreatment of Caco-2 cells with lactobacilli inhibited *S. typhimurium*-induced IL-8 production (<27.3 pg/mL). However, lactobacilli did not inhibit IL-8 production in Caco-2 cells pretreated with *S. typhimurium*. These results suggest that the tested lactobacilli strains are appropriate for preventing inflammatory diseases caused by enteric pathogens but not for therapy. In short, *L. salivarius* and *L. plantarum* are potential candidates for the development of microbial ecological agents and functional foods.

## 1. Introduction

Lactobacilli are natural colonizers of the human gastrointestinal tract (GIT) and a subdominant genus in the colon; therefore, they are generally regarded as safe. *Lactobacillus* has an important function in functional foods and biotherapeutic agents. However, as probiotics, lactobacilli strains must possess certain characteristics to exert their maximum probiotic effects. These potential properties include bacterial adhesion capacity, exclusion of intestinal pathogens, and immunoregulatory effects [1, 2].

The attachment of enteric pathogens to host intestinal epithelial cells (IECs) is an important cause of pathogenesis. The current therapy for intestinal infections is limited to supportive treatment because antibiotic treatment can exacerbate some negative effects. Lactobacilli have been proven to be effective in inhibiting enteropathogenic infections [3, 4] partly by competition with enteric pathogens for enterocyte binding sites, subsequently blocking the attachment of pathogens to IECs [5]. Cell surface properties, such as hydrophobicity and aggregation (autoaggregation and coaggregation), are necessary for the adhesion of probiotics. Probiotics with good surface properties also have potential to establish prior colonization in the GIT and are associated with the exclusion of intestinal pathogens [1, 6].

The secretion of proinflammatory cytokine interleukin-8 (IL-8) by intestinal epithelial cells is mediated by nuclear factor *κ*B (NF-*κ*B) activation in response to certain cytokines and pathogens such as TNF-*α* and *Salmonella typhimurium* [7, 8]. IL-8 can direct inflammatory cell movement into the mucosa, resulting in inflammatory bowel diseases [9]. Recent studies suggest that some lactobacilli strains elicit an anti-inflammatory response [1], whereas some contradictory findings have been reported [10, 11]. Recently, a *L. rhamnosus* GG-derived soluble protein, p40, has been shown to reduce colitis and has beneficial effects on several inflammatory diseases [12]. However, whether cell debris and extracts of lactobacilli, as well as dead lactobacilli cells, modulate the innate immunity is unknown. This ability is important for understanding the mechanism of action of the probiotics and the characteristics of the precise anti-inflammatory molecules.

Previously, we found that two lactobacilli strains, *Lactobacillus salivarius* CICC 23174 and *Lactobacillus plantarum* CGMCC 1.557, have probiotic properties, such as acid and bile tolerance, adhesion capacity, and antibacterial activity (data not published). The aim of the present study is to investigate *in vitro* these two lactobacilli strains and the commercial strain *Lactobacillus rhamnosus* LGG (used as positive control) for their hydrophobicity and their ability to inhibit the attachment of four common intestinal pathogens. We also investigated their ability to modulate IL-8 secretion in Caco-2 cells challenged with TNF-*α* and *S. typhimurium*.

## 2. Materials and Methods

### 2.1. Bacterial Strains and Cell Line

*L. salivarius* CICC 23174 was obtained from the China Center of Industrial Culture Collection (CICC). *L. plantarum* CGMCC 1.557 was obtained from the China General Microbiological Culture Collection (CGMCC). *L. rhamnosus* LGG (ATCC 53103), a well-studied probiotic strain, was purchased from American Type Culture Collection (ATCC) and was used as positive control. *Bacillus cereus* and *S. typhimurium* were isolated from the environment and provided by Dr. Sun from the Academy of the Military Medical Sciences. *Escherichia coli* and *Staphylococcus aureus* were provided by Professor Chen of the Department of Food Science at the Jilin Agricultural University. The human colon adenocarcinoma cell line Caco-2 cells were obtained from the Kunming Institute of Zoology, Chinese Academy of Science.

### 2.2. Bacteria and Caco-2 Cell Culture Conditions

All lactobacilli were cultured in de Man, Rogosa, and Sharpe broth (MRS; Qingdao Hope Bio-Technology Co., Ltd., China) for 14 h to 20 h or on MRS plates (MRS broth supplemented with 1.5% agar; Qingdao Hope Bio-Technology Co., Ltd., China) for 48 h under a 5% CO_2_ atmosphere at 37°C. All pathogens were cultured in Luria-Bertani (LB) medium with shaking for 14 h or on LB plates (LB broth supplemented with 1.5% agar) for 48 h at 37°C.

The Caco-2 cells were grown in Dulbecco's modified Eagle's medium (DMEM; HyClone, Laboratories Inc., Logan, UT, USA) supplemented with 10% heat-inactivated fetal bovine serum (HyClone), l-glutamine (2 mmol/L), penicillin (100 U/mL), and streptomycin (100 mg/mL) in an incubator with 95% (v/v) humidified air and 5% (v/v) CO_2_ at 37°C. Specifically, Caco-2 cells were seeded at a concentration of 1 × 10^5^ cells/mL and subcultured every 4 days. During the culture, the medium was replaced every other day. The cells were used for adhesion study between passages 42 and 65 (cells were passaged at 60% confluence). For the adhesion assays, the Caco-2 cells (passages 42–65) were seeded at 1 × 10^5^ cells/mL (preconfluence) in 24-well tissue culture plates (Corning, Inc., Corning, NY, USA) and fully differentiated for 16 d (postconfluence) by changing the culture medium every 2 d. In the state of postconfluence, Caco-2 cells form a polarised monolayer with typical brush border microvilli. The media of the cells maintained in the confluent state were replaced with fresh unsupplemented DMEM for 1 h prior to the adhesion assay and then rinsed thrice with DMEM.

### 2.3. Measurement of Bacterial Surface Hydrophobicity

Two complementary methods, namely, bacterial adherence to hydrocarbons (BATH) and salt aggregation test (SAT), were performed to assess bacterial surface hydrophobicity.

*BATH [1].* Overnight cultures of the lactobacilli and pathogens were harvested using 10 min of centrifugation at 5000 ×g at room temperature. The pellet was washed twice with sterile PBS (pH 6.7) and then resuspended in 3 mL of 0.1 M KNO_3_ to a final concentration of about 1 × 10^8^ CFU/mL. The mixture was used to estimate its absorbance at 600 nm (A_0_). We mixed 3 mL of the cell suspension with 1 mL of xylene to form a two-phase system. After preincubation at room temperature for 10 min, the two-phase system was mixed by vortexing for 2 min and allowed to stand for 20 min to separate the two phases (water and xylene phases). The aqueous phase was carefully collected and its absorbance was measured at 600 nm (A_1_). All experiments were repeated thrice. Xylene affinity was expressed using the following formula: BATH (%) = (1 − A_1_/A_0_) × 100.

*SAT [13].* Overnight cultures of the lactobacilli and pathogens were harvested using 10 min of centrifugation at 5000 ×g at room temperature. The pellet was washed twice with PBS (0.002 M, pH 6.7) and then resuspended in this buffer to a final concentration of about 1 × 10^8^ CFU/mL. Then, 25 *μ*L of the bacterial suspensions was mixed with equal volumes of ammonium sulfate at various molarities (0.2 M to 4.5 M in 0.002 M PBS (pH 6.7)) in 96-well tissue culture plates. After gentle rotation for 1 min, the lowest ammonium sulfate concentration to cause visual bacterial cell clumping was recorded as the SAT value. The SAT value is inversely proportional to the hydrophobic nature [14].

### 2.4. Coaggregation of Lactobacilli with Pathogens

A coaggregation assay was performed using the method by Collado et al. with minor modifications [15]. Overnight cultures of lactobacilli and pathogen strains were washed twice with PBS (pH 6.7) and resuspended in PBS to a final concentration of 1 × 10^8^ CFU/mL. Equal volumes (1.5 mL) of lactobacilli and pathogen strains were mixed, vortexed for 10 s, and incubated at 37°C for 2 h without agitation. The supernatant liquids were then measured at 600 nm (A_600_). All experiments were repeated thrice. Coaggregation was calculated according to the following equation:

(1) Coaggregation  (%)   =[1−  Amix(Alactobacill+Apathogen/2)]×100,

where A_lactobacill_, A_pathogen_, and A_mix_ represent the A_600_ of lactobacilli, pathogen strains, and their mixture after incubation for 2 h, respectively.

### 2.5. Competition-Based Adhesion Inhibition Assay

A competition-based adhesion assay was performed following a previously reported method with some modifications [2]. Briefly, approximately 1 × 10^5^ Caco-2 cells per well were seeded into a 24-well plate and subcultured at 80% to 90% confluence. The adhesion assays were performed using fully differentiated Caco-2 cells (16 d postconfluence cultures). We cocultured 300 *μ*L of lactobacilli and pathogen (1 × 10^9^ CFU/mL each) suspensions in DMEM (without antibiotics) in each well for 2 h at 37°C under a 5% CO_2_ atmosphere. After incubation, nonadherent cells were discarded by washing thrice with sterile PBS. The cells with adherent bacteria were lysed with 1 mL of Triton X (1%, v/v) for 10 min in an ice-water bath. The pathogens that adhered to Caco-2 cells were serially diluted and spread onto LB agar plates for counting. All experiments were independently performed three times. The adhesion inhibition was calculated according to the following equation:

(2) $$\begin{array}{c} \text{Competitive}\;\text{inhibition}\;\left(\%\right)=\left(1-\frac{N_{\text{probiotic}}}{N}\right)\times 100, \end{array}$$

where *N*_probiotic_ and *N* represent the number of pathogens adhered to Caco-2 cells in the presence and in the absence of probiotic strains, respectively.

### 2.6. Stimulation of Caco-2 Cells by TNF-*α*

Caco-2 cells (1 × 10^5^ cells/well) were seeded into 24-well tissue culture plates and cultured for 48 h. After complete confluence, the Caco-2 cells were washed with sterile PBS (pH 6.7) thrice. The cells were incubated with TNF-*α* (10 ng/mL in DMEM) for 0, 12, 24, and 48 h and with TNF-*α* (2, 4, 6, and 10 ng/mL in DMEM) for 24 h. After coincubation, cell viability was tested via trypan blue exclusion and monolayer integrity, and the supernatants were harvested to quantify IL-8 production using a human IL-8 ELISA Ready-Set-Go kit (eBioscience, San Diego, CA, USA). All experiments were independently performed thrice.

### 2.7. Stimulation of Caco-2 IL-8 Production by Lactobacilli in a Proinflammatory Context

The fully differentiated Caco-2 cells in 24-well plates were washed with sterile PBS (pH 6.7) thrice and then were pretreated with 1 mL of TNF-*α* (10 ng/mL) for 24 h to mimic an inflammatory background. The cells were washed twice with PBS (pH 6.7) and stimulated with live lactobacilli and related preparations for 24 h to assess their anti-inflammatory properties. Caco-2 cells, which were pretreated with TNF-*α* (10 ng/mL) followed by washing and treatment with DMEM, were used as the positive control. After coincubation, the IL-8 concentration in the supernatant was determined using an ELISA kit.

Live lactobacilli and related preparations: the live lactobacilli were used at different concentrations of 1 × 10^7^, 1 × 10^8^, and 1 × 10^9^ CFU/mL in DMEM. The related preparations were made with lactobacilli suspension (1 × 10^9^ CFU/mL in DMEM) through the following procedures: (i) incubation at 65°C or 90°C for 30 min; (ii) antibiotic treatment (100 IU/mL penicillin and 100 *μ*g/mL streptomycin); (iii) preparation of cell extracts and cell debris by sonication, as described by Hwan Choi et al. [16]; (iv) the bacterial cell-free conditioned medium (CM) which was obtained by centrifugation at 4,000 ×g for 10 min, filtered through a 0.22 *μ*m membrane filter (Millipore Co., Cork, Ireland), and stored at −20°C until assay. Caco-2 cells were checked for viability via trypan blue exclusion and monolayer integrity. Separate experiments were performed thrice.

### 2.8. Challenge of Caco-2 Cells with **S. typhimurium ** before and after Treatment with Lactobacilli

The experiment was performed according to the method by Vizoso Pinto et al. with some modifications [10]. Caco-2 cells were pretreated with live lactobacilli and subsequently stimulated with *S. typhimurium*. Caco-2 cells (1 × 10^6^ cells/well) were seeded into 12-well tissue culture plates and cultured for 48 h. After complete confluence, the Caco-2 cells were washed with sterile PBS (pH 6.7) thrice. The cells in each well were pretreated with 1 mL of live lactobacilli (1 × 10^9^ CFU/mL) or *S. typhimurium* (1 × 10^7^ CFU/mL) for 2 h. Then, the cells were washed twice and 1 mL of *S. typhimurium* (1 × 10^7^ CFU/mL) or live lactobacilli (1 × 10^9^ CFU/mL) was added into each well and incubated for another 4 h. All bacterial suspensions used were prepared in DMEM. After incubation, the supernatants were collected to quantify IL-8.

### 2.9. Statistical Analysis

For comparison, one-way ANOVA was performed via a Tukey-Kramer post hoc comparison, and significant differences were assessed using Student's *t*-test. All data are expressed as means ± standard deviation. Statistical analyses were performed using SPSS 14.0 for Windows (SPSS Inc., USA). *P* values less than 0.05 were considered statistically significant.

## 3. Results and Discussion

### 3.1. Bacterial Cell Surface Hydrophobicity

The three lactobacilli strains exerted significantly higher BATH values than the tested pathogens (Table 1). The strains with the highest BATH values were *L. salivarius* (54%) and *L. plantarum* (48%), followed by the commercial strain LGG (36%). The BATH value of pathogens ranged from 9% to 18%. In the SAT assay, all of the tested strains, including lactobacilli and pathogens, exerted similar SAT values.

Bacterial adhesion to IECs is necessary for their colonization in the GIT. This property prevents their elimination by peristalsis and provides an ecological competitive advantage in the GIT [17, 18]. For enteric pathogens, adherence to host IECs is an important step in pathogenesis because this property allows the release of enzymes and toxins that initiate necrotic processes directly into the target cell, consequently causing infection [19, 20]. For lactobacilli, adhesion ability is one of the important criteria for selecting probiotic strains [1, 6] and is involved in the differential modulation of the host immune response [21]. Bacterial adhesion varies among strains, depending on physicochemical properties such as cell surface hydrophobicity [2, 22]. Hydrophobic interaction is a major event in the adherence of bacteria to host cells [23, 24]. Therefore, we first studied the hydrophobicity of lactobacilli and pathogen strains. In hydrophobicity assay, two complementary methods, namely, BATH and SAT, were performed to assess bacterial surface hydrophobicity because reliance on one method for this assay is inadequate [25]. Our results show that although the SAT values of all tested strains were similar, the BATH assay showed that the three lactobacilli strains had significantly higher hydrophobicity (*P* < 0.05) than all of the pathogens tested (Table 1). These results suggests that the three lactobacilli strains have the potential to establish prior colonization in the GIT and are associated with the exclusion of intestinal pathogens [5, 26, 27].

### 3.2. Coaggregation of Lactobacilli with Pathogens

**Figure 1:**
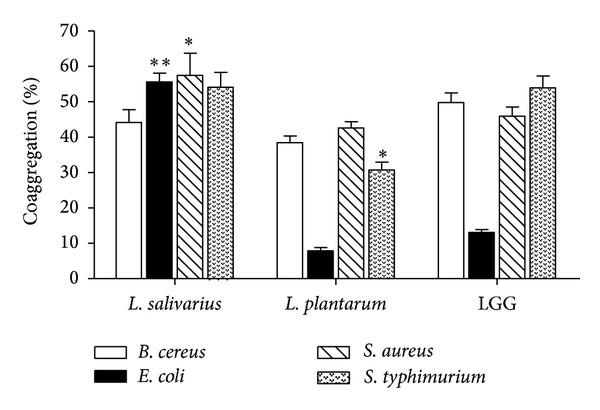
Coaggregation abilities of lactobacilli strains with four pathogens after 2 h incubation at 37°C. Values are presented as means ± SD (*n* = 3). *P < 0.05, **P < 0.01, compared with the *L. rhamnosus* LGG group (control).

All tested lactobacilli strains were highly coaggregated with *B. cereus* (38% to 50%), *S. typhimurium* (31% to 54%), and *S. aureus* (43% to 58%; Figure 1). Among the lactobacilli strains, *L. salivarius* showed the highest coaggregation ability with *E. coli* (56%) and* S. aureus* (57%). LGG showed the least coaggregation abilities with *E. coli* (13%). *L. plantarum* showed the least coaggregation ability with *E. coli* (8%). Thus, *L. salivarius* showed good coaggregation ability with all of the tested pathogens, which ranged from 44% to 57%. *L. plantarum* and LGG showed relatively weak coaggregation properties with *E. coli*, which ranged from 8% to 13%.

Many reports indicated that the coaggregation and autoaggregation abilities of probiotic bacteria help prevent colonization by gut pathogens [28, 29]. Thus, aggregation ability is considered a beneficial property for probiotic strains. We previously showed that *L. salivarius* had higher autoaggregation values (46%) than *L. plantarum* (34%) [30]. In the present study, we found that *L. salivarius* has higher coaggregation values (44% to 57%) than *L. plantarum* (8% to 43%). Our results are in accordance with the studies by Xu and Vlková et al. [2, 29], who suggested that coaggregation is associated with autoaggregation. We also found that *L. salivarius* has higher or similar coaggregation values than the commercial strain LGG. Therefore, *L. salivarius* has the potential to protect host cells from pathogen colonization, preventing intestinal infections.

### 3.3. Inhibitory Effects of Lactobacilli Strains on Pathogen Attachment to Caco-2 Cells

**Figure 2:**
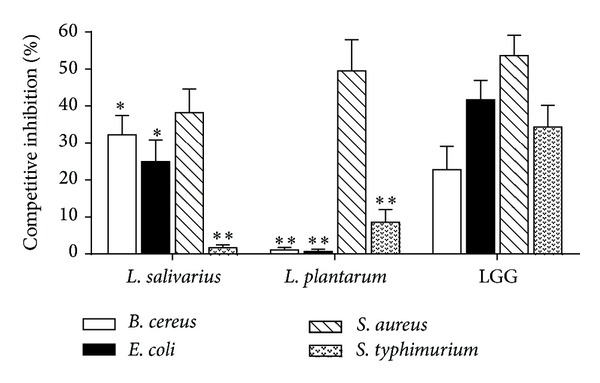
Inhibitory effects of lactobacilli strains on pathogen attachment to Caco-2 cells. The data represent the mean ± SD of three replicates. *P < 0.05, **P < 0.01, compared with the *L. rhamnosus* LGG group (control).

The competitive inhibition of pathogen adhesion to Caco-2 cells by lactobacilli is shown in Figure 2. LGG effectively inhibited the attachment of all tested pathogens (23.0% to 53.6%). *L. salivarius* inhibited the attachment of three pathogens (25.0% to 38.2%) except for *S. typhimurium* (1.7%). *L. plantarum* inhibited only *S. aureus* attachment (49.5%) and exhibited weak inhibitory effects on *B. cereus*,* E. coli*, and *S. typhimurium* attachment (0.7% to 8.6%). *L. salivarius* significantly inhibited *S. aureus* attachment (38.2%).

In Section 3.2, we supposed that *L. salivarius* has the potential to protect host cells from pathogen colonization, preventing intestinal infections. In this section, the competitive adhesion assay supports this hypothesis (Figure 2), which shows that *L. salivarius* is more effective in inhibiting pathogen adhesion to Caco-2 cells than *L. plantarum*.

### 3.4. IL-8 Production in Caco-2 Cells Stimulated with TNF-*α*

**Figure 3:**
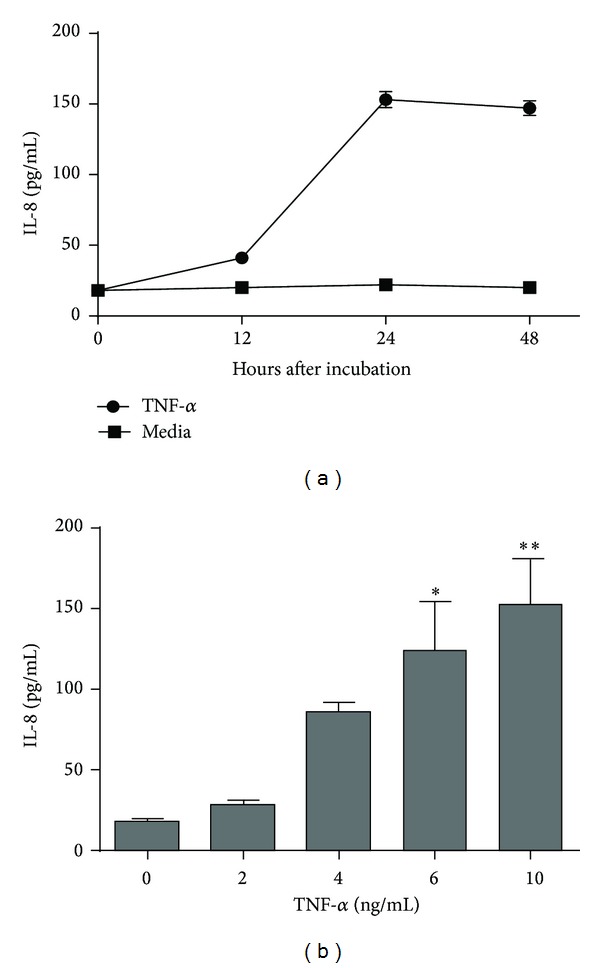
IL-8 production in Caco-2 cells after TNF-*α* stimulation. (a) Time course of response. Caco-2 cells were stimulated with TNF-*α* (10 ng/mL) for various times as indicated. (b) Dose response. Caco-2 cells were stimulated with TNF-*α* at various concentrations as indicated. Unstimulated Caco-2 cells were used as controls. Values are presented as means ± SD (*n* = 3). *P < 0.05, **P < 0.01, compared with the control medium.

TNF-*α* is known to induce the release of the proinflammatory mediator IL-8 from IECs and to activate neutrophils and other inflammatory cells [31]. These activities can cause cell damage and some intestinal bowel diseases. To mimic a proinflammatory background, we determined the time course (Figure 3(a)) and the dose response (Figure 3(b)) of TNF-*α*-induced IL-8 production in Caco-2 cells to establish the optimal time and dose to investigate the inflammatory reaction. Figure 3(a) shows that the Caco-2 cell lines constitutively produced IL-8. After TNF-*α* challenge, IL-8 production increased progressively, peaking (153 pg/mL) at 24 h. Thus, the stimulation time of 24 h was chosen in subsequent IL-8 induction experiments. Then, we investigated the response of Caco-2 cells to various TNF-*α* concentrations. The result shows direct relationships between the IL-8 level and the TNF-*α* dose (Figure 3(b)). At 10 ng/mL, TNF-*α* significantly (*P* < 0.01) stimulated IL-8 production. Therefore, this concentration was chosen in subsequent experiments.

### 3.5. Effect of Lactobacilli on IL-8 Production in Caco-2 Cells Pretreated with TNF-*α*

**Figure 4:**
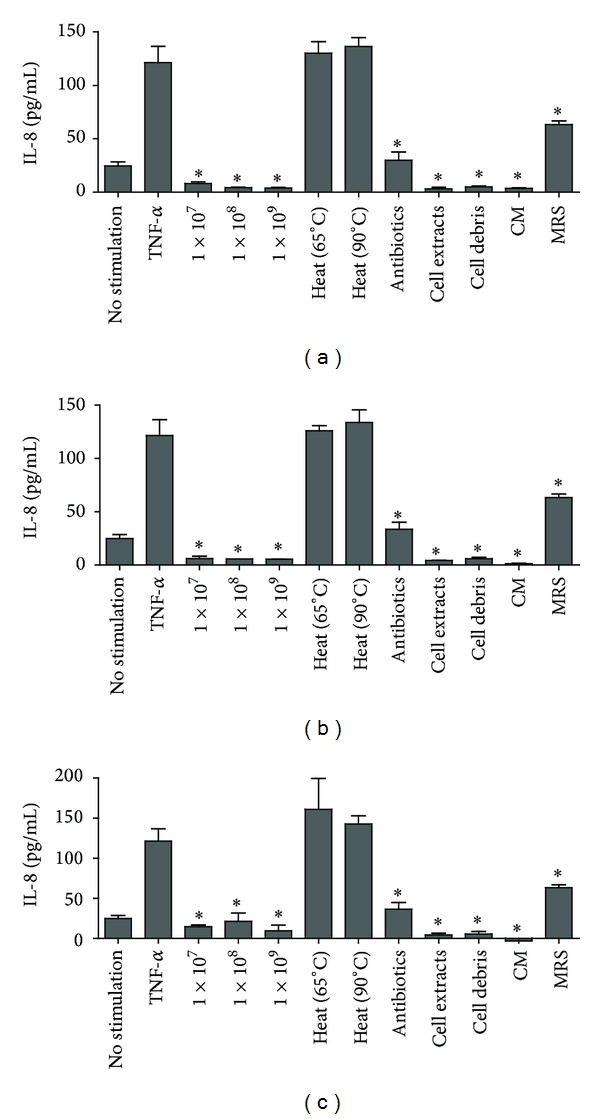
Effect of lactobacilli on IL-8 production by Caco-2 cells in a proinflammatory context. Caco-2 cells were pretreated with TNF-*α* to mimic an inflammatory background and then treated with live lactobacilli (1 × 10^7^, 1 × 10^8^, and 1 × 10^9^ CFU/mL) or related preparations. (a) *L. salivarius*; (b) *L. plantarum*; (c) LGG. Caco-2 cells treated only with TNF-*α* were used as positive controls. Values are presented as means ± SD (*n* = 3). *P < 0.01, compared with TNF-*α*-stimulated cells without lactobacilli treatment. CM: cell-free conditioned medium of lactobacilli; MRS: de Man-Rogosa-Sharpe broth (pH 4.0).

To investigate the response of Caco-2 cells to lactobacilli in an inflammatory context, Caco-2 cells were pretreated with TNF-*α* to mimic an inflammatory background and then treated with live lactobacilli or the related preparations. As shown in Figure 4, TNF-*α* stimulated IL-8 production in the Caco-2 cells up to 121 pg/mL. However, coincubation of the TNF-*α*-stimulated Caco-2 cells with all three lactobacilli strains significantly decreased IL-8 production (*P* < 0.01) in a dose-dependent manner. To investigate further whether the cell components and CM of lactobacilli inhibited IL-8 production, the three lactobacilli strains were treated with heating, antibiotics, sonication, and centrifugation. Exposure of the TNF-*α*-stimulated Caco-2 cells to antibiotic-treated lactobacilli also resulted in a significant decrease in IL-8 production (*P* < 0.01), although this reduction was lower than that obtained with live cells. However, the heat-treated lactobacilli lost their inhibitory effect. Coincubation of the Caco-2 cells with cell debris or lactobacilli cell extract also maintained a similar inhibitory effect compared with that of live whole cells. MRS medium and cell-free conditioned medium of lactobacilli (CM) attenuated TNF-*α*-induced IL-8 production. In the presence of CM, IL-8 production was lower than that obtained with MRS. IL-8 production was not detected in the presence of LGG-CM. The viability of the Caco-2 cells exceeded 95%, and no cell detachment was observed when the cell monolayer was incubated with live lactobacilli or related preparations.

Our findings mentioned above were correlated with previous studies which showed that live lactobacilli and their related preparations reduced the release of IL-8 in the TNF-*α*-pretreated Caco-2 cells [15, 32]. Heat-treated lactobacilli lost their inhibitory effect, whereas antibiotic-treated lactobacilli and sonicated lactobacilli (cell debris and extract) maintained a significant inhibitory effect. These results show that both the structural components and the soluble cellular content of lactobacilli have an important function in anti-inflammatory effects. This observation is consistent with previous studies, which suggested that live probiotics are not necessarily required for anti-inflammatory effects [15, 33, 34]. However, Ma et al. suggested that only live *Lactobacillus reuteri* inhibit TNF-*α*-induced IL-8 production in T84 and HT-29 cells and the inhibitory effect was not reproduced using CM, bacterial lysates, and heat-killed or gamma-irradiated lactobacilli [31]. This discrepancy may have resulted from the different lactobacilli strains used or differences in the Toll-like receptor expression levels in different epithelial cells [1, 10]. These differences suggest that various pathways are associated with the anti-inflammatory effects of lactobacilli.

We also investigated the inhibitory effect of lactobacilli culture supernatant. Our results show that the lactobacilli supernatant inhibited TNF-*α*-induced IL-8 production (Figure 4). This finding is similar to those of other studies, which showed that *Lactobacillus* strains, including *L. rhamnosus*, *Lactobacillus helveticus*, *Lactobacillus casei*, and *L. plantarum*, upregulate anti-inflammatory mediator (IL-10) and downregulate proinflammatory mediators (IL-8 and TNF-*α*) [35–37]. These results imply that the soluble substances secreted by lactobacilli also inhibit TNF-*α*-induced IL-8 production. Previous study reported that two *L. rhamnosus* LGG-derived soluble proteins (p75 and p40) had anti-inflammatory effect [38]. It is not known whether the soluble ingredient from the three lactobacilli strains in this study is the same as the soluble proteins (p75 and p40). Further investigations will be needed to confirm the structural components and the soluble factors of lactobacilli that have an anti-inflammatory effect.

### 3.6. Effect of Lactobacilli on the **S. typhimurium **-Induced IL-8 Production

**Figure 5:**
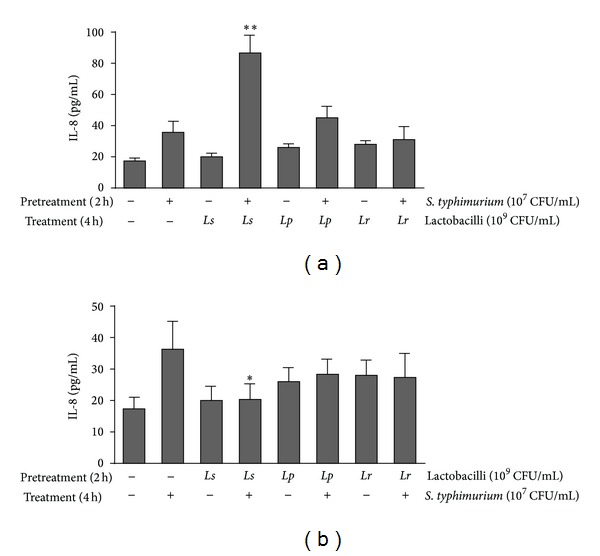
Effect of lactobacilli on IL-8 production by Caco-2 cells before or after *S. typhimurium* infection. (a) Caco-2 cells were pretreated with *S. typhimurium* and then treated with lactobacilli; (b) Caco-2 cells were pretreated with lactobacilli and then treated with *S. typhimurium.* Values are presented as means ± SD (*n* = 3). *P < 0.05, **P < 0.01, compared with the positive control (only *S. typhimurium*-treated group). *Ls*: *L. salivarius*; *Lp*: *L. plantarum*; *Lr*: *L. rhamnosus* LGG.

To investigate the ability of lactobacilli to affect the innate response of Caco-2 cells to *S. typhimurium*, Caco-2 cells were challenged with *S. typhimurium* before and after lactobacilli treatment. When the Caco-2 cells were challenged with *S. typhimurium* alone for 2 h, IL-8 was produced at a higher level (36 pg/mL) than in the unchallenged control (17 pg/mL; Figure 5(a)). After the challenged Caco-2 cells were treated with lactobacilli for a further 4 h, *L. salivarius* significantly increased (*P* < 0.01) the IL-8 level (87 pg/mL). *L. plantarum* also increased the IL-8 level (45 pg/mL), but LGG slightly decreased the IL-8 level (31 pg/mL; Figure 5(a)). However, when the Caco-2 cells were pretreated with lactobacilli for 2 h and then treated with *S. typhimurium* for 4 h, the IL-8 levels were relatively lower than in the Caco-2 cells pretreated without lactobacilli (Figure 5(b)). *L. salivarius* significantly inhibited (*P* < 0.05) the IL-8 production induced by *S. typhimurium*, decreasing from 36 pg/mL to 20 pg/mL.

Our data show that pretreating Caco-2 cells with *S. typhimurium* before lactobacilli treatment does not decrease IL-8 production (Figure 5(a)). However, pretreating Caco-2 cells with lactobacilli before *S. typhimurium* treatment decreases IL-8 production (Figure 5(b)). These results are in accordance with a recent study that showed that *Lactobacillus paracasei* is more effective in inhibiting inflammation in pretreated infected mice than in posttreated infected mice [39]. When lactobacilli were preincubated with Caco-2 cells prior to *S. typhimurium* addition, the lactobacilli inhibited pathogen adhesion by occupying common adhesion sites on Caco-2 cells (barrier effect), modulating the host immune response [26]. IL-8 significantly (*P* < 0.01) increased when the Caco-2 cells were first treated with *S. typhimurium* and then treated with *L. salivarius* (Figure 5(a)). This result suggests that at the tested concentration, this strain is unsuitable for treating inflammatory diseases caused by enteric pathogens.

## 4. Conclusions

In conclusion, this study suggests that *L. salivarius* and *L. plantarum* displayed good cell surface properties, such as hydrophobicity and coaggregation ability. These properties enable the two strains to inhibit the attachment of some pathogens to IECs. Both the structural components and the soluble cellular content of the two lactobacilli strains effectively inhibited TNF-*α*-induced IL-8 production in Caco-2 cells. Both of the lactobacilli strains prevented *S. typhimurium*-induced proinflammatory responses. Therefore, the two selected lactobacilli strains showed probiotic potential for producing microbial ecological agents and functional foods. Further *in vivo* studies are needed to validate their competitive exclusion of pathogens and anti-inflammatory effects, which are currently in progress in our laboratory.

## Figures and Tables

**Table 1:** Surface hydrophobicity of the lactobacilli and pathogen strains.

Bacterial strain	BATH (%)^a^	SAT (M)^b^
*Lactobacillus salivarius *	54 ± 1.8^A^	1.0
*Lactobacillus plantarum *	48 ± 1.4^AB^	2.0
*Lactobacillus rhamnosus *LGG	36 ± 2.1^B^	2.0
*Bacillus cereus *	14 ± 1.3^C^	1.0
*Escherichia coli *	9 ± 0.9^D^	2.5
*Staphylococcus aureus *	18 ± 1.5^C^	1.0
*Salmonella typhimurium *	17 ± 2.1^C^	1.0

^a^BATH: bacterial adherence to hydrocarbons.

^b^SAT: salt aggregation tests.

^A to D^Means with different uppercase superscript letters are significantly different (*P* < 0.05).
